# Prognostic values of blood urea nitrogen/creatinine and cystatin C in patients with radical nephrectomy for renal cell carcinoma

**DOI:** 10.5937/jomb0-45664

**Published:** 2024-06-15

**Authors:** SiCheng Wang, HaoLong Chen, Feng Chao, Jia Bin

**Affiliations:** 1 Heping Hospital Affiliated to Changzhi Medical College, Department of Urology, Changzhi City, China

**Keywords:** radical nephrectomy, blood urea nitrogen, creatinine, cystatin C, prognosis, radikalna nefrektomija, krvni urea nitrati, kreatinin, cistatin C, prognoza

## Abstract

**Background:**

To evaluate the prognostic value of blood urea nitrogen/creatinine ratio (BUN/SCr) and cystatin C (Cys C) in patients with renal cell carcinoma (RCC) after radical nephrectomy.

**Methods:**

The study analysed 348 patients with RCC who underwent radical nephrectomy. The optimal cut-off was obtained based on the ROC of specific survival outcomes and the maximum Youden index. The patients were divided into four groups: Group 1 (low BUN/SCr-low Cys C), Group 2 (low BUN/SCr-high Cys C), Group 3 (high BUN/SCr-low Cys C), and Group 4 (high BUN/SCr-high Cys C). The primary endpoint was cancer-specific survival (CSS), and the secondary endpoint was disease-free survival (DFS).

## Abstract (sr)

**Uvod:**

Cilj ovog istraživanja je bio da se proceni prognostička vrednost odnosa između azota uree i kreatinina (BUN/SCr) u krvi i cistatina C (Cys C) kod pacijenata sa karcinomom bubrega (RCC) nakon radikalne nefrektomije.

**Metode:**

U istraživanju je analizirano 348 pacijenata sa RCC koji su podvrgnuti radikalnoj nefrektomiji. Optimalni prag je određen na osnovu ROC krive za specifične ishode preživljavanja i maksimalnog Youden indeksa. Pacijenti su podeljeni u četiri grupe: Grupa 1 (nizak BUN/SCr - nizak Cys C), Grupa 2 (nizak BUN/SCr - visok Cys C), Grupa 3 (visok BUN/SCr - nizak Cys C) i Grupa 4 (visok BUN/SCr - visok Cys C). Primarni krajnji ishod je bio preživljavanje specifično za karcinom (CSS), a sekundarni krajnji ishod bio je preživljavanje bez bolesti (DFS).

**Rezultati:**

Pokazana je snažna pozitivna korelacija između vrednosti BUN/SCr i nivoa Cys C. Pacijenti sa višim odnosom BUN/SCr (17,41) i nivoom Cys C (3,98 mg/L) su imali lošije ishode preživljavanja. Primetno je da su pacijenti u grupi 4 pokazali najlošije stope CSS i DFS, dok pacijenti u grupama 1 i 2 imaju bolje ishode preživljavanja bez značajne razlike između ove dve grupe. Viši odnos BUN/SCr (17,41) i visok nivo seruma Cys C (3,98 mg/L) su bili nezavisni prediktori za CSS i DFS, pored veličine tumora pre operacije i patološkog T (pT) stadijuma.

**Zaključak:**

Ovo istraživanje pruža prve dokaze o nezavisnom prognostičkom značaju odnosa BUN/SCr i Cys C kod pacijenata sa RCC nakon radikalne nefrektomije.

## Introduction

Renal cell carcinoma (RCC) accounts for over 90% of kidney cancers [1]. According to the World Health Organization’s new classification of kidney tumours, clear cell RCC (ccRCC) accounts for most RCC cases [2]. Operative radical nephrectomy (ORN) has been the gold standard for malignant renal masses for decades [3]. In addition, ORN is the standard surgical procedure for T3 and T4 tumours [4]. ORN involves the entire kidney and surrounding perirenal fat, ipsilateral adrenal glands, and regional lymph nodes [5]. The mortality rate of RCC still accounts for a high proportion of malignant urinary system tumours, and the course and prognosis of individual diseases are uneven and difficult to predict [6]. The ability to accurately predict the prognosis of cancer patients concerning death is critical to determining treatment and monitoring strategies for patients. An accurate understanding of disease progression and risk of death is essential to guide patients, plan individualized surveillance protocols, and select appropriate treatment plans. In this case, tumour size, histological subtype, pathological stage, and nuclear grade have been identified as prognostic factors for RCC [7].

The blood urea nitrogen (BUN)/creatinine (SCr) ratio is associated with disease severity and survival. A survey of 139 COVID-19 patients shows that high BUN/SCr predicts severe disease and poor survival [8]. Epidemiological studies have found a bidirectional relationship between renal cancer and impaired renal function [9] [10]. A bidirectional causal relationship has been determined between kidney cancer and renal function biomarkers, including BUN/SCr ratio [11]. Cystatin C (Cys C) is widely expressed in early organisms and involved in immune-related processes under pathological conditions [12]. Urinary Cys C is a potential early marker of acute kidney injury [13] and a valuable prognostic indicator in malignant tumours [14] [15].

However, due to eradicating the primary tumour, major surgery may affect the host’s immune response and kidney function. No studies have investigated BUN/SCr ratio and Cys C when evaluating RCC patients’ prognosis after ORN. In this case, we investigated the prognostic significance of BUN/SCr ratio and serum Cys C in patients undergoing ORN.

## Materials and methods

### Study population

The Institutional Review Board of the Heping Hospital, affiliated with Changzhi Medical College, approved the current study. We reviewed the medical records of 462 patients with RCC diagnosed and treated in Heping Hospital, affiliated with Changzhi Medical College, between January 2012 and January 2018. We included only 368 patients with no lymph nodes and distant metastases and who had underundergone ORN. Patients with the following history were excluded: (1) prior history of any malignancy, a second primary cancer diagnosed before treatment; (2) Postoperative SCr, Cys C, and BUN data were incomplete. Finally, 348 patients were enrolled in the study.

### Research design

Clinicopathologic variables included age at surgery, gender, body mass index (BMI), tumour location, tumour size, histological type, pT stage, and Fuhrman nuclear grade. The eighth edition of UICC/AJCC categorizes pathology according to the tumour-node-metastasis (TNM) system. Postoperative blood samples were collected within 3 months after surgery. All patients underwent a disease history consult, physical examination, routine laboratory tests, and imaging tests such as chest X-rays and computed tomography of the kidneys. Follow-up assessments of all patients were conducted every 3 months for the first 2 years and then every 6 months according to our institution’s protocol until January 2023.

The primary endpoint was cancer-specific survival (CSS), and the secondary endpoint was diseasefree survival (DFS). Survival results were examined via the utilization of receiver operating characteristic (ROC) curve analysis, wherein the optimal cut-off value is determined through ROC analysis (Table 1). We divided the patients into 4 groups based on the threshold value: Group 1 (low BUN/SCr-low Cys C), Group 2 (low BUN/SCr-high Cys C), Group 3 (high BUN/SCr-low Cys C), and Group 4 (high BUN/SCrhigh Cys C).

**Table 1:** Cut-off and corresponding sensitivity and specificity based on maximum Youden Index.

		Cut-off	Sensitivity<br>%	Specificity<br>%
Cancer-specific<br>survival	BUN/<br>Scr ratio	17.41	49.62	90.91
	Cys C<br>(mg/L)	3.98	47.96	80.91
Disease-free<br>survival	BUN/Scr<br>ratio	14.91	57.71	73.61
	Cys C<br>(mg/L)	3.14	59.50	72.2

### Sample collection and measurement

The experimental participants were instructed to collect venous blood from the elbow vein after fasting [12], allow it to coagulate at room temperature, and separate the serum within 4 hours of collection. The serum was then tested at a constant temperature of 4 using a fully automated biochemical analyser (ADVIA2400, SIEMENS AG, Berlin, Germany) to analyse BUN, SCr, and Cys C. Shanghai Kehua Biotechnology Co., Ltd., manufactured the Cys C kit used in the analysis and was strictly operated in accordance with the instructions provided with the kit. The normal range for BUN was determined to be 3.8 to 7.0 mmol/L; for SCr, it was 45 to 105 μmol/L; and for Cys C, it was 0.70 to 1.15 mg/L.

### Data analysis

All statistical analyses were conducted by SPSS software 22.0. The chi-square test was used for classification parameters, and the Mann-Whitney U test for continuous parameters. Spearman correlation coefficient was applied to analyse the correlation between serum factors. The AUC was calculated using ROC analysis, and the optimal critical value was selected based on the maximum Youden index. The Youden index is sensitivity plus specificity minus 1, and the larger the Youden index, the higher the accuracy of diagnosis. The survival rate was analysed by Kaplan-Meier and tested by logarithmic rank test. In addition, univariate and multivariate Cox regression models were employed to analyse the correlation between variables and survival further. Bilateral *P*-values represented statistical significance at <0.05.

## Results

### Patient characteristics

This study summarized the demographics of 348 patients with RCC who underwent ORN (not shown in the table). The median age was 56 years (IQR, 47–66 years), and 66.7% were male. The median BUN was 7.8 mmol/L (IQR, 7.0∼9.1 mmol/L), SCr was 131.6 μmol/L (IQR, 114.1∼140.3 μmol/L), BUN/SCr ratio was 14.80 (IQR, 13.83–16.58 mg/L), and Crs C was 3.06 mg/L (IQR, 2.02–3.98 mg/L). The median BMI was 23.3 kg/m^2^ (IQR, 21.2–26.7 kg/m^2^).

Table 2 shows the baseline characteristics of patients according to BUN/SCr values and serum Cys C cut-off. The number (proportion) of patients in each classification is as follows: There were 300 cases (15.41%) (BUN/SCr ratio<17.41), 48 cases (13.8%) (BUN/SCr ratio≥17.41), 267 cases (76.7%) (Cys C<3.98 mg/L), and 81 cases (23.2%) (Cys C≥3.98 mg/L). We observed that patients with higher BUN/SCr ratio (>17.41) and high Cys C level (>3.98 mg/L) had a higher proportion of tumour size over 7 cm than patients with lower BUN/SCr ratio and Cys C (*P*=0.010, *P*=0.008).

**Table 2:** Cut-off and corresponding sensitivity and specificity based on maximum Youden Index.

Variable	BUN/SCr ratio		p value	Cys C		p value
No. of patients (%)	<17.41300(86.2)	≥17.4148(13.8)		<3.98267(76.7)	≥3.9881(23.2)	
Age (years)						
<60	121(40.3)	26(54.2)	0.072	126(47.2)	33(40.7)	0.307
≥60	179(59.7)	22(45.8)		141(52.8)	48(59.3)	
Gender						
Female	99(33.0)	17(35.4)	0.742	189(70.8)	49(60.5)	0.081
Male	201(67.0)	31(64.6)		78(29.2)	32(39.5)	
BMI (kg/m^2^)						
<25	179(59.7)	34(70.8)	0.140	159(59.6)	61(75.3)	0.010
≥25	121(40.3)	14(29.2)		108(40.5)	20(24.7)	
Tumour site						
Left	160(53.3)	21(43.8)	0.217	140(52.4)	40(49.4)	0.630
Right	140(46.7)	27(56.2)		127(47.6)	41(50.6)	
Tumour size (cm)						
<7	238(79.3)	30(62.5)	0.010	210(78.6)	52(64.2)	0.008
≥7	62(20.7)	18(37.5)		57(21.4)	29(35.8)	
Histoloy						
Clear cell	246(82.0)	41(85.4)	0.563	215(80.5)	70(86.4)	0.227
Others	54(18.0)	7(14.6)		52(19.5)	11(13.6)	
pT classification						
pT1-pT2	236(78.7)	32(66.7)	00.67	212(79.4)	56(69.1)	0.054
pT3-pT4	64(21.3)	16(33.3)		55(20.6)	25(30.9)	
Fuhrman grades						
G1-G2	91(30.3)	11(22.9)	0.295	85(31.8)	20(25.9)	0.220
G3-G4	209(69.7)	37(77.1)		182(68.2)	61(74.1)	
BUN (mmol/L)	7.1[6.1∼8.1]	8.8[8.0∼10.1]	<0.001	7.9[7.3∼8.9]	9.4[8.4∼10.7]	<0.001
SCr (mmol/L)	135.1[107.2∼138.6]	133.1[115.4∼140.2]	0.09	135.3[115.3∼140.8]	131.7[122.3∼138.4]	0.324
Cys C (mg/L)	2.60[1.98∼3.43]	4.41[4.19∼4.52]	<0.001	2.54[1.95∼3.24]	4.19[4.12∼4.45]	<0.001

**Figure 1:**
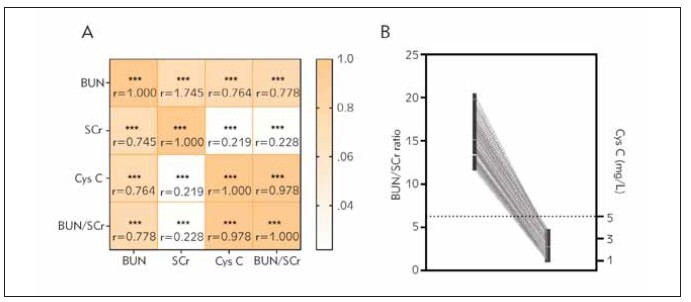
Correlation analysis results. Correlation between BUN, SCr, BUN/SCr and Cys C in patients’ serum (A); Double axis line chart (B). Spearman’s correlation coefficient is calculated, where r is the correlation coefficient. *** p<0.001; ** p<0.01; * p<0.05.

For other clinicopathological variables, no significant differences were observed. It was worth noting that serum Cys C significantly differed between the group with high BUN/SCr and low BUN/SCr (*P*<0.001). Serum BUN significantly differed between the groups with high and low Cys C levels (*P*<0.001). This suggests that there seems to be a correlation between serum BUN and Cys C, which was confirmed by Spearman correlation analysis (r=0.764, *P*<0.001) in Figure 1A. We also found a stronger positive correlation between BUN/SCr ratio and Cys C (r=0.978, *P*<0.001) (Figure 1A, Figure 1B).

### Correlation between BUN/SCr ratio, serum Cys C, and prognosis

**Figure 2:**
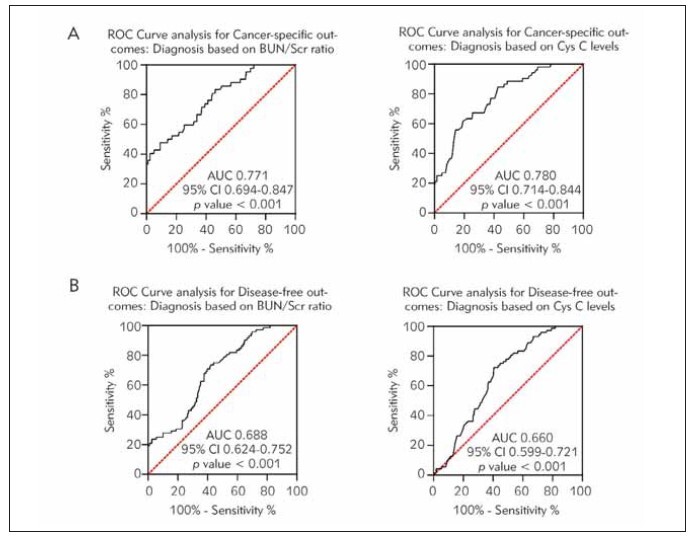
ROC curves for evaluating BUN/SCr ratio and Cys C. CSS (A); DFS (B). p<0.05.

During a mean follow-up of 75.9 months, disease-specific mortality was 11.2% (n = 39), and all-cause mortality was 12.6% (n=44). Twenty-eight patients (8.05%) experienced cancer recurrence. The AUC for specific survival results based on BUN/SCr ratio or serum Cys C level was 0.771 (95%CI=0.694–0.847, *P*<0.001) and 0.780 (95%CI=0.714–0.844, *P*<0.001), respectively (Figure 2A). The AUC for DFS was 0.688 (95%CI=0.624–0.752, *P*<0.001) and 0.660 (95%CI=0.599–0.721, *P*<0.001), respectively (Figure 2B). The critical value, sensitivity, and specificity of each ROC curve are shown in Table 1.

**Figure 3:**
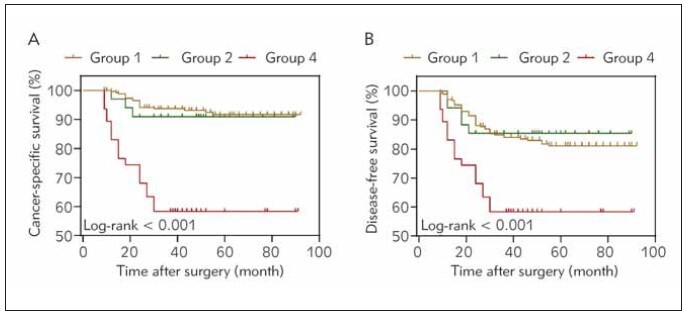
Kaplan-Meier curve analysis results. Specific survival time (A); DFS for subgroups (B). p<0.05.

Next, groups were set according to the critical value, and Group 3 (high BUN/SCr-low Cys C) had only one patient, so this group was not analysed. Kaplan-Meier survival analysis showed (Figure 3) that patients in Group 4 (high BUN/SCr-high Cys C) had poorer CSS and DFS than those in Group 1 (low BUN/SCr-low Cys C) and Group 2 (low BUN/SCr-low Cys C). No significant difference in survival outcomes was observed between Groups 1 and 2. After adjusting for various clinicopathological factors, we finally determined that BUN/SCr ratio and serum Cys C were predictors of CSS and DFS in multivariate Cox analysis, in addition to tumour size and pT stage (Table 3 and Table 4).

**Table 3:** Univariate and multivariate analyses of predictors of cancer specific survival for patients with renal cell carcinoma who underwent radical nephrectomy.

Variables	Univariate			Multivariate		
	HR	95% CI	P value	HR	95% CI	P value
Age (years)						
<60<br>≥60	0.96	0.56–1.76	0.856			
Gender						
Male<br>Female	0.85	0.72–2.35	0.341			
BMI (kg/m^2^)						
<25<br>≥25	0.48	0.35–0.84	0.024	0.89	0.45–2.23	0.145
Tumour size<br>(cm)<br><7<br>≥7	4.652	2.45–7.68	<0.001	3.561	1.89–6.99	<0.001
pT classification						
pT1-pT2<br>pT3-pT4	2.12	1.13–3.58	0.001	1.96	1.03–3.41	0.015
Fuhrman grades						
G1-G2<br>G3-G4	1.68	0.98–3.25	0.096			
BUN/SCr ratio						
<17.41<br>≥17.41	2.32	1.84–5.62	0.005	2.01	1.76–5.30	0.023
Cys C (mg/L)						
<3.98<br>≥3.98	2.85	2.03–5.76	<0.001	2.59	1.88–5.60	0.001

**Table 4:** Univariate and multivariate analyses of predictors of Disease free survival for patients with renal cell carcinoma who underwent radical nephrectomy

Variables	Univariate			Multivariate		
	HR	95% CI	P value	HR	95% CI	P value
Age (years)						
<60<br>≥60	1.22	0.75–2.38	0.352			
Gender						
Male<br>Female	1.32	0.62–2.45	0.385			
BMI (kg/m^2^)						
<25≥25	0.59	0.35–0.84	0.024	0.82	0.45–2.03	0.526
Tumour size (cm)<br><7<br>≥7	3.89	2.02–6.78	<0.001	3.26	1.59–6.09	<0.001
pT classification						
pT1-pT2<br>pT3-pT4	2.01	1.06–3.38	0.001	2.06	1.14–3.01	0.012
Fuhrman grades						
G1-G2G3-G4	1.56	0.92–3.13	0.079			
BUN/SCr ratio						
<17.41<br>≥17.41	1.89	1.46–4.23	0.010	1.56	1.26–3.39	0.033
Cys C (mg/L)						
<3.98<br>≥3.98	1.76	1.23–3.02	0.001	1.42	1.19–3.01	0.016

## Discussion

This was the first study to show that survival outcomes in RCC patients undergoing ORN were significantly associated with BUN/SCr and Cys C. In particular, patients with higher BUN/SCr and Cys C had worse CSS and DFS results than patients with lower BUN/SCr and Cys C. More importantly, among patients with high BUN/SCr and Cys C, 19 deaths occurred, all of which were cancer-specific, accounting for 40.4%. However, no significant differences were found in CSS and DFS results between group 1 (low BUN/SCr and low Cys C) and group 2 (low BUN/SCr and high Cys C). BUN/SCr and Cys C were predictors of survival outcomes.

Patients with RCC have decreased renal function after surgery [16] [17]. Although the mechanism is unclear, ORN is potentially associated with chronic kidney disease in patients with renal cancer [18]. In a follow-up of 500 patients with ORN, the cumulative 5-year incidence of chronic kidney disease was 43.4% [19]. Chronic kidney disease can develop into end-stage renal disease, and patients have an increased risk of death [20]. After ORN, the glomerular filtration rate is significantly reduced, suggesting impaired renal function [21]. BUN/SCr is associated with the prognosis of patients with heart failure or renal impairment caused by acute infarction. For example, BUN/Cr exceeding the optimal threshold could predict the disease condition and poor survival of more severe COVID-19 patients [8]. Moreover, BUN/Cr is associated with long-term mortality from heart failure [22].

Cys C has been determined as a marker of renal insufficiency or kidney injury [23]. The use of Cys C avoids limitations related to diet, nutrition, and muscle mass that affect BUN and SCr [24]. In addition, Cys C can provide reliable prognostic information for diseases after treatment, such as kidney transplantation [25], heart transplantation [26], and percutaneous coronary intervention for acute myocardial infarction [27].

Our results showed that BUN/SCr had a strong positive correlation with Cys C. The combination of low BUN/SCr and low Cys C accounted for 76.4%, high BUN/SCr and high Cys C 13.5%, low BUN/SCr and high Cys C 9.8%, and high BUN/SCr and low Cys C 1 case. This suggests that combining the two may have strong prognostic information for patients after surgery. In our study, some patients below the threshold survived, while some above it died, and vice versa. Nevertheless, the patient-specific mortality in Group 4 (high BUN/SCr with high Cys C) was 40.4%, compared to 7.1% in Group 1. This is similar to some previous reports that the detection of BUN/SCr or Cys C is associated with a relatively poor prognosis [15] [28]. High BUN/SCr values and high Cys C serum levels after ORN were also highly predictive and independent predictors of mortality risk. Our analysis showed that the threshold of 17.41 (BUN/SCr), 3.89 mg/L (Cys C) is the most appropriate level to distinguish between specific survival and death. When this threshold is exceeded, the specific mortality of the population increases by 1.8 to more than 5 times. Similarly, exceeding the threshold is a risk factor for DFS. Although we did not analyse whether BUN/SCr combined serum Cys C was an independent risk factor for death, these data suggest that high BUN/SCr values and high serum Cys C levels may strongly predict patient outcomes even after surgery.

The current study has some potential limitations. First, this is a retrospective study and has inevitable limitations, including the lack of consistent data collection, relatively small sample size, and high percentage of missing data in some groups. Second, our data are based on single-centre trials. Therefore, external validation is needed to substantiate the results of the current study further. Finally, we could not provide mechanistic clues to support our new findings on the prognostic effects of postoperative BUN/SCr values and Cys C levels on survival outcomes in patients with RCC. Nevertheless, we demonstrated independence from established prognostic factors, including tumour size and pathological T stage. We reported that high BUN/SCr values combined with high Cys C levels were associated with a risk of poor prognosis in patients with RCC undergoing ORN.

## Dodatak

### Funding

Not applicable.

### Data availability statement

The data that support the findings of this study are available from the corresponding author upon reasonable request.

### Ethical statement

All procedures performed in studies involving human participants were in accordance with the ethical standards of Heping Hospital Affiliated to Changzhi Medical College and with the 1964 Helsinki declaration and its later amendments or comparable ethical standards.

### Conflict of interest statement

All the authors declare that they have no conflict of interest in this work.

## References

[1] Hsieh J J, Purdue M P, Signoretti S, Swanton C, Albiges L, Schmidinger M, et al (2017). Renal cell carcinoma. Nat Rev Dis Primers.

[2] Jenei A, Hes O, Kuthi L J A K (2020). Provisional renal cell carcinoma subsets following the 2016 WHO classification.

[3] Tabbara M M, González J, Ciancio G (2023). The surgical evolution of radical nephrectomy and tumor thrombectomy: A narrative review. Ann Transl Med.

[4] Jeon S H, Kwon T G, Rha K H, Sung G T, Lee W, Lim J S, et al (2011). Comparison of laparoscopic versus open radical nephrectomy for large renal tumors: A retrospective analysis of multi-center results. BJU Int.

[5] Robson C J, Churchill B M, Anderson W (2017). The results of radical nephrectomy for renal cell carcinoma. J Urol.

[6] Čechová M, Chocholatý M, Babjuk M, Kalousová M, Zima T (2022). Prognostic factors of renal cell carcinoma. Rozhl Chir: mesicnik Ceskoslovenske chirurgicke spolecnosti.

[7] Klatte T, Rossi S H, Stewart G D (2018). Prognostic factors and prognostic models for renal cell carcinoma: A literature review. World J Urol.

[8] Ok F, Erdogan O, Durmus E, Carkci S, Canik A (2021). Predictive values of blood urea nitrogen/creatinine ratio and other routine blood parameters on disease severity and survival of COVID-19 patients. J Med Virol.

[9] Kong H J, Park J S, Kim D Y, Shin H S, Jung H J (2013). Renal function following curative surgery for renal cell carcinoma: Who is at risk for renal insufficiency?. Korean J Urol.

[10] Krebs R K, Andreoni C, Ortiz V (2014). Impact of radical and partial nephrectomy on renal function in patients with renal cancer. Urol Int.

[11] Lin Y, Yang Y, Fu T, Lin L, Zhang X, Guo Q, et al (2023). Impairment of kidney function and kidney cancer: A bidirectional Mendelian randomization study. Cancer Med.

[12] Guo K, Chen Q, He X, Yao K, Li Z, Liu Z, et al (2018). Expression and significance of Cystatin-C in clear cell renal cell carcinoma. Biomed Pharmacother.

[13] Bagshaw S M, Bellomo R (2010). Cystatin C in acute kidney injury. Curr Opin Crit Care.

[14] Leto G, Crescimanno M, Flandina C (2018). On the role of cystatin C in cancer progression. Life Sci.

[15] Nückel H, Langer C, Herget-Rosenthal S, Wichert M, Assert R, Döhner H, et al (2012). Prognostic significance of serum cystatin C in multiple myeloma. Int J Hematol.

[16] Singla N, Hutchinson R, Menegaz C, Haddad A Q, Jiang L, Sagalowsky A I, et al (2016). Comparing changes in renal function after radical surgery for upper tract urothelial carcinoma and renal cell carcinoma. Urology.

[17] Ohno Y, Nakashima J, Ohori M, Hashimoto T, Iseki R, Hatano T, et al (2011). Impact of tumor size on renal function and prediction of renal insufficiency after radical nephrectomy in patients with renal cell carcinoma. Urology.

[18] Coyle D, Quinlan M R, D'Arcy F T, Kelly B D, Corcoran O, Durkan G C, et al (2015). Pattern of change in renal function following radical nephrectomy for renal cell carcinoma. Ir Med J.

[19] Wang S, Liu Z, Zhang D, Xiang F, Zheng W (2022). The incidence and risk factors of chronic kidney disease after radical nephrectomy in patients with renal cell carcinoma. BMC cancer.

[20] Vejakama P, Ingsathit A, McEvoy M, Attia J, Thakkinstian A (2017). Progression of chronic kidney disease: An illness-death model approach. BMC Nephrol.

[21] Miyamoto K, Inoue S, Kajiwara M, Teishima J, Matsubara A (2012). Comparison of renal function after partial nephrectomy and radical nephrectomy for renal cell carcinoma. Urol Int.

[22] Parrinello G, Torres D, Testani J M, Almasio P L, Bellanca M, Pizzo G, et al (2015). Blood urea nitrogen to creatinine ratio is associated with congestion and mortality in heart failure patients with renal dysfunction. Intern Emerg Med.

[23] Fu Z, Xue H, Guo J, Chen L, Dong W, Gai L, et al (2013). Long-term prognostic impact of cystatin C on acute coronary syndrome octogenarians with diabetes mellitus. Cardiovasc Diabetol.

[24] Ferguson T W, Komenda P, Tangri N (2015). Cystatin C as a biomarker for estimating glomerular filtration rate. Curr Opin Nephrol Hypertens.

[25] Lezaic V, Dajak M, Radivojevic D, Ristic S, Marinkovic J (2014). Cystatin C and serum creatinine as predictors of kidney graft outcome. Int Urol Nephrol.

[26] Franeková J, Hošková L, Sečnik P, Jr, Pazderník M, Kotrbatá M, Kubíček Z, et al (2016). The role of timely measurement of galectin-3, NT-proBNP, cystatin C and hsTnT in predicting prognosis and heart function after heart transplantation. Clin Chem Lab Med.

[27] Shen G, Zhu H, Ding H, Sun C, Zhou K, Fan Y, et al (2018). Increased cystatin C level in ST-elevation myocardial infarction predisposes the prognosis of angioplasty. Am J Med Sci.

[28] Lin H L, Chen C W, Lu C Y, Sun L C, Shih Y L, Chuang J F, et al (2012). High preoperative ratio of blood urea nitrogen to creatinine increased mortality in gastrointestinal cancer patients who developed postoperative enteric fistulas. Kaohsiung J Med Sci.

